# Do interpersonal trust and social avoidance mediate the association between psychotic symptoms and social functioning in chronic schizophrenia patients?

**DOI:** 10.3389/fpsyt.2025.1433763

**Published:** 2025-03-25

**Authors:** Hongyang Liu, Xinming Pan, Xinxin Huang, Haijia Tian, Xueke Shao, Dandan Wang, Lu Wen, Chenxi Bao, Xinyu Fang, Zhenghui Yi

**Affiliations:** ^1^ The Affiliated Wenzhou Kangning Hospital, Wenzhou Medical University, Zhejiang Provincial Clinical Research Center for Mental Disorders, Wenzhou, China; ^2^ Department of Psychiatry, The Second People’s Hospital of Jiangning District, Nanjing, China; ^3^ Department of Psychiatry, The Affiliated Brain Hospital of Nanjing Medical University, Nanjing, China; ^4^ Shanghai Mental Health Center, Shanghai Jiao Tong University School of Medicine, Shanghai, China; ^5^ Institute of Mental Health, Fudan University, Shanghai, China; ^6^ Department of Psychiatry, Huashan Hospital, Fudan University, School of Medicine, Shanghai, China

**Keywords:** schizophrenia, psychotic symptoms, interpersonal trust, social avoidance, social functioning

## Abstract

**Objective:**

Schizophrenia is a complex mental disorder that significantly impacts social functioning. The present study aimed to investigate the relationship between psychotic symptoms and social functioning in individuals with chronic schizophrenia. Specifically, we examined the mediating roles of social avoidance and impaired interpersonal trust in this relationship, as these factors are most worrisome in individuals with schizophrenia.

**Methods:**

A total of 223 outpatients with chronic schizophrenia and 201 unrelated healthy controls were included. The Positive and Negative Syndrome Scale (PANSS), the Interpersonal Trust Scale (ITS), the Social Avoidance and Distress Scale (SAD), and the simplified Chinese version of the Social Disability Screening Schedule (SDSS) were used for evaluation. Mediation analysis was performed using the PROCESS macro in SPSS23.0.

**Results:**

Our results showed that individuals with chronic schizophrenia scored significantly lower on the ITS total and two index scores but higher on the SAD total and two index scores than healthy controls. The ITS score was significantly associated with the psychotic symptoms (both PANSS total score and subscale score) and social functioning in those patients. Interestingly, we further found that interpersonal trust had a significantly mediating effect on the relationship between psychotic symptoms (including positive, negative, cognitive, excited, and depressed symptoms) and social functioning in individuals with schizophrenia.

**Conclusion:**

Our preliminary findings suggest that improving interpersonal trust may be a promising approach to enhance social functioning and improve prognosis in individuals with schizophrenia. This insight underscores the importance of incorporating trust-building interventions into clinical practice, which could potentially lead to better social outcomes for patients.

## 1 Introduction

Schizophrenia is a severe mental illness that mainly characterized by positive symptoms (e.g., hallucinations, delusions, disorganized speech, and abnormal movements), negative symptoms (such as flattened affect, asociality, anhedonia, apathy, and lack of emotions), general pathology symptoms (e.g., depressive and anxious mood) and cognitive impairment (including attention, processing speed, memory, executive functioning, and social cognition) (1–3). It affects 1% of the world population and places huge economic burdens on patients, their families, and society as a whole (4). Currently, antipsychotic drugs are the main treatment for schizophrenia, which often have a good effect on improving positive symptoms but have little effect on negative symptoms and cognitive impairment (5). Moreover, negative symptoms and cognitive impairment are usually associated with poor social performance and personal functions, thus leading to a poor prognosis in these patients. Additionally, existing literature has demonstrated that positive and negative symptom severity or the lack of symptom improvement are crucial risk factors related to poor treatment compliance in individuals with schizophrenia (6). Given the adverse effects of psychiatric symptoms on functional outcomes in patients with schizophrenia, it is essential to identify and intervene in potential mediating factors that may influence this relationship and thus provide benefits to individuals with schizophrenia.

Building on this understanding, interpersonal trust emerges as a critical factor that could mediate the relationship between psychiatric symptoms and functional outcomes. Interpersonal trust has been described as a core dimension of cooperative, mutually beneficial interpersonal relationships and the basis for the development of a concept of fairness and moral identity among children and adolescents (7). It plays an essential role in almost all human relationships and is an essential behavior for developing and maintaining supportive social relationships and social interaction with others (8). The lack of interpersonal trust is significantly associated with poor social interaction and social avoidance (9), and such social avoidance can further reduce the potential benefits of social situations and result in problematic behavior and difficulty adapting to changes (10). Given the importance of interpersonal trust for adaptive psychosocial functioning, deficits in interpersonal trust have also been regarded as a risk factor for psychopathology in adolescence (11). Prior studies have observed significant associations between interpersonal trust and conduct disorder, suicide, externalizing problems, and delinquency (12, 13). Moreover, ample evidence has demonstrated that interpersonal trust beliefs are strongly associated with mental illness, including depression (14), borderline personality disorder (11, 15), autism spectrum disorder (16), and schizophrenia (17). Previous studies employing the trust game have demonstrated reduced trust in individuals with schizophrenia (8, 18). Moreover, existing evidence has shown that interpersonal trust is also reduced in individuals at clinical high risk for psychosis and in healthy first-degree relatives of those with schizophrenia who have increased genetic risk for schizophrenia (19, 20). The above findings suggest that reduced trust is related to the risk for psychosis and may be an endophenotype of schizophrenia (20). Hence, more studies should be conducted to understand interpersonal trust in schizophrenia and explore its relationship to psychotic symptoms and their impact on functional outcomes in those patients.

To date, there are surprisingly few studies that have examined the link between interpersonal trust and psychotic symptoms in individuals with schizophrenia, especially in chronic patients. An early study included 39 adolescents with early psychosis and found that higher negative symptoms were significantly associated with lower interpersonal trust in the trust game, but there was no significant association between interpersonal trust and positive symptoms in those patients (21). Interestingly, another study with 24 schizophrenia patients suggested that the lack of interpersonal trust is attributable to their positive symptoms (17). Since there are limited and contradictory results, more research involving larger samples of patients with schizophrenia is warranted to further examine and clarify these relationships. In addition, whether reduced interpersonal trust presents in individuals with chronic schizophrenia should also be investigated.

Additionally, the relationships between interpersonal trust and social avoidance and social function in individuals with schizophrenia deserve considerable attention. Social avoidance is commonly seen in individuals with schizophrenia (22), and it was hypothesized that social avoidance may be a consequence of a diminished anticipatory pleasure or distress in response to psychotic experiences (23, 24). A recent study conducted among college students documented that interpersonal trust closely interacted with social avoidance (9). Although substantial evidence supports that both interpersonal trust and social avoidance are predictors of adverse functional outcomes in schizophrenia (25–27), no study to date has explored the relationship between interpersonal trust and social avoidance in individuals with schizophrenia. Given the prevalence of interpersonal trust and social withdrawal in individuals with schizophrenia, clarifying the complex relationship between psychotic symptoms, interpersonal trust, social withdrawal, and social functioning in individuals with schizophrenia may help improve their prognosis.

Therefore, the purpose of the present study aimed to investigate: (1) whether patients with chronic schizophrenia experienced difficulties in interpersonal trust and social avoidance compared to healthy controls; (2) whether interpersonal trust is significantly correlated with psychotic symptoms, social avoidance and social functioning in these patients; and (3) whether interpersonal trust and social avoidance affect the relationship between psychotic symptoms and social functioning in these patients. It was hypothesized that individuals with chronic schizophrenia have reduced interpersonal trust and increased social avoidance compared to healthy controls and that interpersonal trust and social avoidance could mediate the role of psychotic symptoms on social functioning in individuals with schizophrenia.

## 2 Methods

### 2.1 Subjects

We recruited 223 chronic outpatients with schizophrenia from psychiatric hospitals (Wenzhou Kangning Hospital and the Second People’s Hospital of Jiangning District, Nanjing). The criteria for selecting the participants were as follows: (1) meeting the diagnosis of schizophrenia based on the Structured Clinical Interview for DSM-5 (SCID) by two psychiatrists; (2) Han Chinese, aged 20–65 years; (3) had a disease duration of ≥ 36 months (28) and received fixed-dose antipsychotic treatment for at least 6 months prior to this study. The exclusion criteria were as follows: (1) had physical comorbidities, such as head trauma or intellectual disability; (2) had comorbid substance abuse or dependence; (3) refusal to participate in the study. A total of 201 unrelated healthy controls who were age and sex matched were recruited from the local community and underwent the SCID to exclude the presence of a current/past mental disorder. Each participant signed a written informed consent form, which was approved by the Institutional Ethical Committee for Clinical Research of Wenzhou Kangning Hospital. All procedures were conducted in strict accordance with the Declaration of Helsinki.

### 2.2 Clinical assessment

The Positive and Negative Syndrome Scale (PANSS) is a 30-item clinician-rated scale to measure the severity of the psychotic symptoms of individuals with schizophrenia. The five-factor model, including the positive (items P1, P3, P5, G9), negative (items N1, N2, N3, N4, N6), cognitive/disorganized (items P2, N5, G11), excited (items P4, P7, G8, G14), and depressed (items G2, G3, and G6) factors, was used in the present study based on the previous literature and guidance from the European Psychiatry Association (29, 30). Two psychiatrists who received training sessions conducted the face-to-face interviews and maintained an inter-rater correlation coefficient greater than 0.8.

Interpersonal trust was assessed using the Interpersonal Trust Scale (ITS), which was developed by Rotter in 1967. It is a self-reporting tool consisting of 25 items on a 5-point scale (ranging from 1 = totally agree to 5 = totally disagree). Higher scores indicate higher amounts of trust, and factor analysis found two factors in the Chinese version of the ITS: trust in relatives and friends (special trust factor) and trust of indirectly related people (general trust factor) (31).

Social avoidance was evaluated using the Social Avoidance and Distress Scale (SAD) (Watson and Friend, 1969). It consists of 28 items, of which 14 are used to rate social avoidance and 14 are used to rate social distress. Items are self-rated on a “Yes–No” scale, with total scores ranging from 0 to 28. The total score reflects the tendency to avoid social interaction and the distress experienced as a result of social interaction, with higher scores indicating greater social discomfort and avoidance tendencies. The α-coefficient in this study was greater than 0.75, which indicated high reliability and validity.

The simplified Chinese version of the Social Disability Screening Schedule (SDSS) was used to measure the social, occupational, and psychological functioning of the patients. Each item was rated from 0 to 2, with a total score ranging from 0 to 20. Higher scores reflect worse social functioning. It works well in social functioning evaluations of individuals with schizophrenia and has been administered with good validity and reliability among Chinese samples (32).

### 2.3 Statistical analysis

Statistical analysis was performed using the Statistical Package for the Social Sciences, version 23.0 (SPSS 23.0). The significance value was set at P < 0.05 in all two-tailed tests. We divided individuals with schizophrenia into a high interpersonal trust group and a low interpersonal trust group according to the mean score of the ITS of all patients. For the between-group comparison of demographic and clinical characteristics, the chi-squared test for categorical variables and the independent-sample t-test for continuous variables were applied. An analysis of covariance (ANCOVA) was used to control for confounding variables. Further, the Pearson correlation was performed to examine the correlation between clinical variables in the patients with schizophrenia and healthy controls. Bonferroni corrections were used for multiple tests. Finally, we tested whether interpersonal trust mediated the relationship between psychotic symptoms and social functioning in individuals with schizophrenia using the PROCESS macro in SPSS23.0. A standard procedure was followed using bootstrap sampling with 5,000 iterations, which produced 95% bias-corrected confidence intervals (CIs). The mediation effect was statistically significant if the bias-corrected CI did not include 0.

## 3 Results

### 3.1 Comparisons between chronic schizophrenia and healthy controls

**Table 1** shows the demographic and clinical characteristics of the patients with chronic schizophrenia and healthy controls. There were no significant differences in age, sex, and height between the patients and controls (all P > 0.05). We found that individuals with schizophrenia had higher weights (t = 4.356, P < 0.001) but lower marriage rates (X^2^ = 36.924, P < 0.001) compared to the healthy controls, thus, the weight and marital status were controlled for in the following analysis. Our results showed that the individuals with schizophrenia scored significantly lower on the ITS total and two index scores but higher on the SAD total and two index scores than the healthy controls (all P < 0.001). After controlling for age, sex, weight, and marital status, all these significant differences still remained in the multivariate analysis of covariance (all P < 0.001).

**Table 1:** Comparisons between schizophrenia patients and healthy controls.

	Healthy controls (N = 201)	Schizophrenia (N = 223)	t/X^2^	P
Age (year)	37.30 ± 9.75	37.98 ± 9.91	0.714	0.475
Sex			0.335	0.563
Male	98	115		
Female	103	108		
Height (cm)	167.02 ± 22.22	166.56 ± 7.63	0.290	0.772
Weight (Kg)	61.80 ± 12.38	67.11 ± 12.69	4.356	<0.001
Marital status			36.924	<0.001
Unmarried	75	149		
Married	126	74		
SAD total score	5.04 ± 1.89	12.52 ± 3.39	28.434	<0.001
Social distress	2.61 ± 1.41	6.33 ± 2.47	19.245	<0.001
Social avoidance	2.43 ± 1.19	6.18 ± 1.89	24.727	<0.001
ITS total score	99.55 ± 7.17	60.52 ± 15.10	34.515	<0.001
Special trust factor	51.08 ± 4.51	29.11 ± 9.73	30.309	<0.001
General trust factor	48.47 ± 4.44	31.41 ± 8.26	26.830	<0.001

Data are presented as Mean ± Standard deviation or Number.

ITS, the Interpersonal Trust Scale; SDSS, the Social Disability Screening Schedule,

### 3.2 Comparisons between high interpersonal trust and low interpersonal trust patients

**Table 2** shows the demographic and clinical characteristics of the high interpersonal trust and low interpersonal trust patients (107 vs. 116). There were no significant differences in age, sex, height, weight, marital status, family history, and age of onset between these two groups (all P > 0.05). Compared to the low interpersonal trust patients, we found that the patients with high interpersonal trust had lower total score (t = 6.185, P < 0.001), and lower scores for the positive symptom subscale (t = 2.809, P = 0.005), negative symptom subscale (t = 5.126, P < 0.001), and general psychopathology subscale (t = 5.829, P < 0.001) of the PANSS. In addition, the high interpersonal trust patients had better social functioning (t = 10.698, P < 0.001) compared to the low interpersonal trust patients. After controlling for age, sex, and marital status, the differences in PANSS total and subscale scores, the ITS total and subscale score, and SDSS between these two patient groups remained significant (all P < 0.05). Curiously, our results found no significant differences in SAD total score and the social distress subscale and social avoidance subscale scores between these two patient groups (all P > 0.05).

**Table 2:** Comparisons between high interpersonal trust and low interpersonal trust patients.

	Low trust group (N = 116)	High trust group (N = 107)	t/X^2^	P
Age (year)	37.18 ± 9.27	38.85 ± 10.54	1.258	0.210
Sex			2.437	0.118
Male	54	61		
Female	62	46		
Height (cm)	166.93 ± 8.41	166.15 ± 6.69	0.770	0.442
Weight (Kg)	68.02 ± 13.40	66.13 ± 11.85	1.116	0.266
Marital status			0.021	0.885
Unmarried	77	72		
married	39	35		
Family history			2.352	0.125
No	88	90		
Yes	28	17		
Age of onset (year)	24.22 ± 6.87	24.64 ± 7.91	0.424	0.672
PANSS	59.08 ± 12.07	48.24 ± 14.07	6.185	<0.001
Positive factor	7.84 ± 3.99	6.45 ± 3.21	2.870	0.005
Negative factor	12.04 ± 5.30	9.50 ± 4.34	3.939	<0.001
Cognitive/disorganized factor	6.05 ± 2.68	4.94 ± 2.33	3.282	0.001
Excited factor	5.89 ± 1.95	5.41 ± 1.89	1.852	0.065
Depressed factor	6.07 ± 2.43	4.38 ± 1.93	5.762	<0.001
SDSS	18.47 ± 4.23	11.86 ± 4.98	10.698	<0.001
SAD total score	12.55 ± 3.17	12.48 ± 3.62	0.165	0.869
Social distress	6.24 ± 2.34	6.43 ± 2.62	0.568	0.571
Social avoidance	6.31 ± 1.67	6.05 ± 2.10	1.040	0.299
ITS total score	48.46 ± 8.28	73.60 ± 8.44	22.443	<0.001
Special trust factor	22.10 ± 4.15	36.70 ± 8.22	16.526	<0.001
General trust factor	26.35 ± 7.30	36.90 ± 5.16	12.526	<0.001

Data are presented as Mean ± Standard deviation or Number.

PANSS, the Positive and Negative Syndrome Scale; ITS, the Interpersonal Trust Scale; SDSS, the Social Disability Screening Schedule.

### 3.3 Correlation analysis

Our results showed that ITS total score was negatively associated with the PANSS positive factor score (r = -0.199, P = 0.003), negative factor score (r =-0.270, P < 0.001), cognitive factor score (r =-0.245, P < 0.001), excited factor score (r =-0.152, P < 0.023) and total score (r = -0.414, P < 0.001), and SDSS score (r = -0.653, P < 0.001) in all patients. All the correlations remained significant (P_Bonferroni_ < 0.05) after the Bonferroni correction was performed except for the correlation between the ITS total score and PANSS excited factor score in the patients (P_Bonferroni_ > 0.05). There was no significant correlation between interpersonal trust and social avoidance in both the patients and healthy controls (all P > 0.05). Additionally, our results showed that PANSS total score, and positive factor, negative factor, cognitive factor, and excited factor subscale scores were all positively correlated with SDSS score (PANSS: r = 0.417, P < 0.001; positive factor: r = 0.291, P < 0.001; negative factor: r = 0.256, P < 0.001; cognitive factor: r = 0.282, P < 0.001; excited factor: r = 0.161, P = 0.016,; depressed factor: r = 0.386, P < 0.001) in the patients. The correlations between PANSS total and subscale score and SDSS score remained significant after the Bonferroni correction was performed (All P_Bonferroni_ < 0.05). There were no significant correlations between social avoidance and either psychotic symptoms or social functioning in the patients with schizophrenia (All P > 0.05).

### 3.4 Mediating model

**Figure 1:**
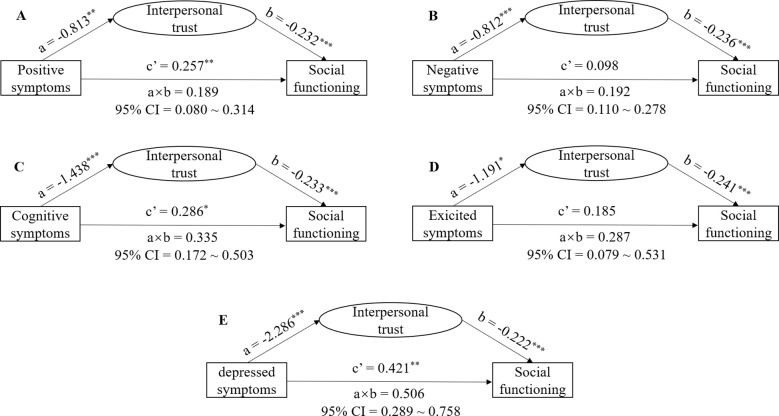
Hypothesized mediation model: indirect effect of interpersonal trust on the relationship between psychotic symptoms and social functioning in individuals with chronic schizophrenia. **(A)** PANSS positive factor-ITS-SDSS model; **(B)** PANSS negative factor-ITS-SDSS model; **(C)** PANSS cognitive factor-ITS-SDSS model; **(D)** PANSS excited factor-ITS-SDSS model; **(E)** PANSS depressed factor-ITS-SDSS model. * P <0.05, **P <0.001. Path a is independent variable (X) → mediator (M). Path b is mediator (M) → dependent variable (Y), adjusted for X. Path c’ is X → dependent variable (Y), adjusted for M. Path a×b is X→ Y through M. * p < 0.05, ** p < 0.01, *** p < 0.001.

According to the principle of mediation effect, both the PANSS subscale score and ITS total score were significantly associated with SDSS. Hence, we performed a medication analysis to test whether interpersonal trust could mediate the effect of psychotic symptoms on social functioning in individuals with chronic schizophrenia (**Figure 1**). However, since the correlation analyses did not reveal any significant correlations between social avoidance and either psychotic symptoms or social functioning, this suggested that social avoidance cannot mediate the impact of psychotic symptoms on social functioning in schizophrenia, so we did not proceed with the mediation model involving psychotic symptoms, social avoidance and social functioning.

In the PANSS positive factor-ITS-SDSS model, we found significant direct effects of the PANSS positive factor on SDSS [c’ = 0.257, P = 0.001, 95% CI (0.103, 0.410)], the PANSS positive factor on ITS [a = -0.813, P = 0.003, 95% CI (-1.344, -0.283)], and ITS on SDSS (b = -0.232, P < 0.001, 95% CI = -0.270, -0.195). The total effect of the PANSS positive factor on SDSS was 0.446 [P < 0.001, 95% CI (0.251, 0.640)]. The indirect effect of the PANSS positive factor through ITS on SDSS was 0.189 (95% CI [0.080, 0.313]). The results indicated that interpersonal trust partially mediates the effect of the PANSS positive factor on social functioning in schizophrenia (with a 57.6% direct effect and a 42.4% indirect effect). In the PANSS negative factor-ITS-SDSS model, the direct effect of the PANSS negative factor on SDSS was 0.098 [P = 0.102, 95% CI (-0.019, 0.215)], the PANSS negative factor on ITS was -0.812 [P < 0.001, 95% CI (-1.196, -0.427)], and ITS on SDSS was -0.236 (P < 0.001, 95% CI = -0.275, -0.197). The total effect of the PANSS negative factor on SDSS was 0.289 [P = 0.001, 95% CI (0.145, 0.434)]. The indirect effect of the PANSS negative factor through ITS on SDSS was 0.192 [95% CI (0.110, 0.278)]. The results indicated that interpersonal trust partially mediates the effect of the PANSS negative factor on social functioning in schizophrenia (with a 33.8% direct effect and a 66.2% indirect effect). In the PANSS cognitive factor-ITS-SDSS model, we found significant direct effects of the PANSS cognitive factor on SDSS [c’ = 0.286, P = 0.013, 95% CI (0.068, 0.511)], the PANSS cognitive factor on ITS [a = -1.438, P = 0.001, 95% CI (-2.192, -0.684)], and ITS on SDSS (b = -0.233, P < 0.001, 95% CI = -0.271, -0.195). The total effect of the PANSS cognitive factor on SDSS was 0.621 [P < 0.001, 95% CI (0.341, 0.901)]. The indirect effect of the PANSS cognitive factor through ITS on SDSS was 0.335 [95% CI (0.172, 0.503)]. The results indicated that interpersonal trust partially mediates the effect of the PANSS cognitive factor on social functioning in schizophrenia (with a 46.1% direct effect and a 53.9% indirect effect). In the PANSS excited factor-ITS-SDSS model, the direct effect of the PANSS excited factor on SDSS was 0.185 [P = 0.221, 95% CI (-0.112, 0.483)], the PANSS excited factor on ITS was -1.191 [P = 0.023, 95% CI (-2.216, -0.167)], and ITS on SDSS was -0.241 (P < 0.001, 95% CI = -0.279, -0.203). The total effect of the PANSS excited factor on SDSS was 0.473 [P = 0.016, 95% CI (0.089, 0.856)]. The indirect effect of the PANSS excited factor through ITS on SDSS was 0.287 [95% CI (0.079, 0.531)]. The results indicated that interpersonal trust partially mediates the effect of the PANSS excited factor on social functioning in schizophrenia (with a 39.2% direct effect and a 60.8% indirect effect). In the PANSS depressed factor-ITS-SDSS model, we found significant direct effects of the PANSS depressed factor on SDSS [c’ = 0.421, P = 0.001, 95% CI (0.168, 0.674)], the PANSS depressed factor on ITS [a = -2.286, P < 0.001, 95% CI (-3.081, -1.492)], and ITS on SDSS (b = -0.222, P < 0.001, 95% CI = -0.261, -0.182). The total effect of the PANSS depressed factor on SDSS was 0.928 [P < 0.001, 95% CI (0.634, 1.222)]. The indirect effect of the PANSS depressed factor through ITS on SDSS was 0.506 [95% CI (0.289, 0.758)]. The results indicated that interpersonal trust partially mediates the effect of the PANSS depressed factor on social functioning in schizophrenia (with a 45.4% direct effect and a 54.6% indirect effect).

## 4 Discussion

To the best of our knowledge, this is the first study that aimed to compare interpersonal trust and social avoidance between individuals with chronic schizophrenia and healthy controls and to examine the relationship between interpersonal trust, social avoidance, psychotic symptoms, and social functioning in these patients. The main findings of the present study include: (1) Individuals with chronic schizophrenia had reduced interpersonal trust but increased social avoidance compared to healthy controls. (2) Interpersonal trust was significantly associated with the PANSS positive and negative factors, general psychopathology, and social functioning in all individuals with schizophrenia. (3) Interpersonal trust had a partial mediating effect on the relationship between psychotic symptoms (including positive, negative, cognitive, excited, and depressed symptoms) and social functioning in these patients.

In the present study, our results showed that patients with chronic schizophrenia had reduced interpersonal trust compared to healthy controls, which was also reported in patients with first-episode psychosis, individuals at clinical high risk for psychosis, and first-degree relatives of patients with schizophrenia (20, 33). Impaired interpersonal trust in different stages of schizophrenia suggests that it may be an endophenotype of schizophrenia. However, other studies reported the absence of a difference in interpersonal trust between patients with psychosis and controls (34). Since the previous studies had small sample sizes, a recent meta-analysis included 183 patients with psychosis and 272 controls and further demonstrated significant trust deficits in patients with psychosis (35). It is supposed that several social cognitive processes, especially mentalizing processes (referring to the ability to infer, implicitly or explicitly, the intentions, dispositions, and beliefs of others) (36), might result in altered interpersonal trust in patients with psychosis, since evidence supports that intact social cognition is crucial in decision-making and modification of trust behavior (37). Interestingly, impairments in social cognition were widely reported in individuals with schizophrenia (38), and a recent experiment demonstrated that altered oxytocin levels in the lateral parietal cortex and amygdala play an important role in regulating interpersonal trust among individuals with schizophrenia, as measured through a social cognition task (39). Despite some preliminary findings, the exact mechanisms of how social cognition affects interpersonal trust in schizophrenia remain unclear, and further studies are warranted to unravel this issue.

Psychotic symptoms in patients with schizophrenia were also correlated with interpersonal trust, but with mixed results. One study reported that higher PANSS positive and negative symptoms were associated with lower interpersonal trust in early psychosis (34), but another research only indicated a significant correlation between negative symptoms and interpersonal trust in patients with psychosis (21). Moreover, a recent study demonstrated that only positive symptoms are significantly associated with reduced trust in individuals with schizophrenia (17). In another study, researchers failed to reveal any association between psychotic symptoms and interpersonal trust in individuals with schizophrenia (40). In the current study, we recruited a relatively large sample of individuals with schizophrenia and utilized the five-factor PANSS. Our results indicated that reduced interpersonal trust was significantly associated with the severity of positive, negative, cognitive, excited, and depressed symptoms in individuals with chronic schizophrenia. The discrepancies in findings regarding the relationship between different dimensions of psychotic symptoms and interpersonal trust can be attributed to a combination of factors, including sample characteristics, disease state, measurement tools, and study design. Future research should aim to address these factors to provide more consistent and reliable findings. Despite the discrepancies, there is still considerable evidence supporting the relationship between the different dimensions of psychotic symptoms and interpersonal trust in schizophrenia. Paranoia and delusions of being persecuted are the most commonly observed positive symptoms in individuals suffering from schizophrenia. Thinking that others intend to harm them might lead to defensive aggressive behaviors as self-protection or avoiding normal interaction with others, and this weakens the ability to develop interpersonal trust in individuals with schizophrenia (19, 41). The relationship between negative symptoms, depressed symptoms, and reduced interpersonal trust may be reflected by the lack of social motivation. Individuals with schizophrenia with negative symptoms rarely engage in normal social interaction and usually evaluate other persons in a more negative way and lack interpersonal trust (42). However, no study has explored the relationship between cognitive and excited factors and interpersonal trust in schizophrenia since most existing studies did not use the five-factor PANSS (17, 34).

Unsurprisingly, we found that positive, negative, cognitive, excited, and depressed symptoms were significantly associated with social functioning in individuals with schizophrenia, which was also observed in many previous studies (43–45). We know that the social function deficits in schizophrenia significantly impact their daily life, rehabilitation work, and return to society (46). Although the negative symptoms are among the strongest independent predictors of social functioning in individuals with schizophrenia (44), some studies have indicated that negative symptoms influence real-world functioning while also correlating with positive symptoms in schizophrenia (45). Moreover, an early study demonstrated that the negative symptoms of blunted affect and passive-apathetic social withdrawal and the positive symptoms of hallucinatory behavior and suspiciousness predicted social functioning in individuals with schizophrenia, but the total scores on these clinical rating scales include other variables that actually dilute the prediction of functional outcomes in the patients (43). In the current study, our goal was not to split the items of these scales but to explore the effect of interpersonal trust on the relationship between psychotic symptoms and social functioning in individuals with chronic schizophrenia, and we tested our hypothesis. It is supposed that trusting others and returning the trust placed in oneself with trustworthy actions are important aspects of everyday interactions, and the impairment in this fundamental ability can lead to poor social functioning in individuals (35). In the present study, our results further revealed that interpersonal trust could aggravate the influence of psychotic symptoms on social functioning in individuals with chronic schizophrenia. As such, it is important that we develop interventions to improve interpersonal trust in individuals with schizophrenia to promote their social functioning.

In the present study, our results showed that individuals with chronic schizophrenia had significant social avoidance compared to healthy controls. Social avoidance in young patients is always a clinically worrisome phenomenon and often readily contributes to the onset of schizophrenia. Both retrospective studies of first episode patients and prospective studies of patients at risk for psychosis supported that social decline occurred up to several years before the first episode of schizophrenia (47, 48). In addition, ample evidence indicates that individuals with chronic schizophrenia also have significant social withdrawal (22, 49), and the current study further supports this point.

Although a recent study revealed a significant correlation between social withdrawal and interpersonal trust in the general population (50), we failed to find this relationship in individuals with schizophrenia. Since few researchers have explored this issue, further studies are warranted to investigate the relationship between interpersonal trust and social avoidance in individuals with schizophrenia and to better understand their effects on function and prognosis in those patients.

Therefore, treatment of psychotic symptoms through antipsychotic drugs and non-pharmacological treatments is the first step toward improvement of social functioning in individuals with schizophrenia. Meanwhile, our findings suggest that enhancing interpersonal trust in these patients can further improve their social functioning and assist in their successful reintegration into society. Hence, future studies should focus on exploring effective approaches to enhance interpersonal trust among individuals with schizophrenia.

There are some limitations in the present study. First, the cross-sectional nature of the research precludes us from identifying causal relationships among psychotic symptoms, interpersonal trust, and social functioning in individuals with schizophrenia. Second, the ITS and SAD are both self-report evaluations, and individuals with schizophrenia tend to exhibit severe cognitive impairment and lack of judgement and insight (51, 52), potentlly affecting the accuracy of their self-reported interpersonal trust and social avoidance. Additionally, the Interpersonal Trust Scale may yield different results compared to behavioral tools such as the trust game. Thus, our results should be further validated using behavioral measures to ensure their robustness. Third, PANSS may not be the most suitable tool for evaluating negative symptoms in schizophrenia since it includes some aspects that are not conceptualized as negative symptoms and only focuses on the patient’s behavior but fails to assess the subject’s internal experience. Fourth, the study sample was outpatients and thus may not extend to inpatients. Finally, some other factors that are commonly associated with social functioning, such as neurocognition and side effects of drugs, were not collected in the current study. Therefore, the findings in the current study should be considered preliminary, and future studies with a comprehensive longitudinal design and objective measures should be carried out to replicate our findings.

To conclude, the results indicated that individuals with schizophrenia had reduced interpersonal trust and increased social avoidance compared to healthy controls, and interpersonal trust had a mediating role between psychotic symptoms and social functioning in individuals with schizophrenia. It provides a new perspective for the clinical promotion of social functioning and prognosis in individuals with schizophrenia, that is, from the perspective of improving their interpersonal trust. The preliminary findings warrant further exploration and verification.

## Data Availability

The raw data supporting the conclusions of this article will be made available by the authors, without undue reservation.
